# The relationship between parental mental health and early childhood caries: a meta-analysis and systematic review

**DOI:** 10.1038/s41405-026-00429-w

**Published:** 2026-04-22

**Authors:** Lili Ma, Liying Jia, Kaixuan Han, Yanyan Zhang, Shu Fan, Hong Shi

**Affiliations:** 1 Department of Pediatric Dentistry, Hospital of Stomatology and Hebei Provincial Key Laboratory of Stomatology, Hebei Medical University, Shijiazhuang, China; 2 Department of Preventive Dentistry, Hospital of Stomatology and Hebei Provincial Key Laboratory of Stomatology, Hebei Medical University, Shijiazhuang, China

**Keywords:** Paediatric dentistry, Enamel

## Abstract

**Objectives:**

This study aimed to systematically evaluate the relationship between parental mental health and early childhood caries (ECC), providing scientific basis for researching the etiology and enabling clinical prediction and prevention of ECC.

**Materials and methods:**

A comprehensive search was conducted across five databases to identify relevant studies published up to October 2025. Eligible studies were those investigating the relationship between parental mental health and ECC. Data extraction and synthesis were performed following PRISMA guidelines using Stata 16.0. Robustness, heterogeneity, certainty of evidence, and publication bias were evaluated.

**Results:**

Out of 2889 studies, 12 were included in the meta-analysis. From 12 included studies, 3 explored parental depression, 7 explored parental dental anxiety, 4 explored parental stress, and 1 explored parental general anxiety disorder. The meta-analysis results showed that parental mental conditions increased the risk of ECC (ECC prevalence rate: OR = 1.54, 95%CI: 1.12-2.12) and (dmft: SMD = 0.19, 95%CI: 0.13-0.26), significantly.The subgroup results showed that parental dental anxiety and depression were both associated with ECC (OR = 1.59, 95% CI: 1.00-2.51; OR = 1.49, 95% CI: 1.09-2.04). There was no relationship between parental stress and ECC (OR = 2.19, 95%CI: 0.36-13.20). The meta-analysis results of adjustment ORs about parental stress were similar to the crude ORs (OR = 0.81, 95%CI: 0.58-1.14).

**Conclusions:**

The results indicated a strong association between parental mental conditions and ECC. Furthermore, higher levels of dental anxiety and depression in parents were associated with a higher prevalence of ECC in their children. However, the relationship between psychological stress and ECC was not observed. Results must be interpreted with caution due to the limitations of the evidence base.

## Introduction

Early childhood caries (ECC) is a severe chronic childhood disease, especially in developing countries.The American Academy of Pediatric Dentistry (AAPD) defines ECC as the presence of one or more decayed (non-cavitated or cavitated), missing (as a result of caries), or filled tooth surfaces in any primary tooth in a child 71 months of age or younger [1]. The 2018 International Association of Pediatric Dentistry (IAPD) report founded that the global prevalence of ECC among children aged 1-5 years was 17% (1-year-olds), 36% (2-year-olds), 43% (3-year-olds), 55% (4-year-olds), and 63% (5-year-olds) [2]. The Fourth National Oral Health Epidemiological Survey of China revealed that the caries prevalence rates among 3, 4 and 5-year-old children were 50.8%, 63.6%, and 71.9% respectively. The average number of decayed, missing, and filled teeth (dmft) was 2.28, 3.40, and 4.24 [3]. Although, the prevalence of ECC is high, whereas its treatment rate remains extremely low. As of 2018, the dental caries treatment rates for children aged 3, 4, and 5 in China were only 1.5%, 2.9%, and 4.1%, respectively [3]. ECC adversely affects both the development and eruption of successor permanent teeth and children’s physical-psychological well-being [4, 5]. Therefore, the prevention and treatment of ECC has become a critical issue impacting children’s health, and represents a particularly severe challenge for pediatric dentists.

ECC is primarily a sugar-induced dysbiosis of the dental biofilm, leading to intra- and extracellular polysaccharide production, biofilm accumulation, and acid generation, which in turn causes reversible subsurface demineralization and, potentially, cavitation [6]. From a non-biological perspective, substantial evidence confirmed that parental mental health is a risk factor for ECC [7–21]. For example, parents experiencing chronic stress may have reduced capacity to maintain their children’s oral hygiene or may provide diets higher in sugar, ultimately increasing the child’s caries risk [15, 16, 18].

Mental disorders ranked among the top ten causes of global disease incidence in the Global Burden of Disease Study (GBD) 2019, emerging as a growing public health concern worldwide [22]. According to World Health Organization (WHO) in 2019, approximately 970 million people were affected by mental disorders, with anxiety and depression representing the most prevalent conditions [23]. Beyond compromising patients’ own oral health [24], these disorders disrupt offspring cognition and behavior [8]. Studies have reported up to 40% of mothers experience depressive symptoms during pregnancy [17, 25], which can interfere with the growth and development of offspring. A cross-sectional study conducted by Gavic et al. [7] found that parental levels of anxiety, depression, and psychological stress were associated with ECC. Quiñonez et al. [14] observed that children of parents with high stress levels also had a higher prevalence of ECC. Seow et al. [26] concluded that mothers in the ECC group had higher scores for depression and anxiety than those in the caries-free group. However, in contrast to these findings, Alade et al.’s study [8] demonstrated that maternal generalized anxiety disorder, depression, dental anxiety, and psychological stress were not significantly correlated with ECC prevalence. The association between parental mental health and ECC remains inconclusive.

The purpose of this systematic review and meta-analysis was to assess the available evidence regarding the relationship between parental mental health and ECC. We hypothesized that children of parents with mental conditions would be more likely to develop ECC than those of mentally healthy parents.

## Materials and methods

### Protocol and registration

This systematic review was conducted by the standards of the Preferred Reporting Items for Systematic Reviews and Meta-analysis (PRISMA) 2020 statement [27]. It was registered at the International Prospective Register of Systematic Reviews (PROSPERO), registration number CRD42024561778.

### Eligibility criteria

Two reviewers identified and selected potentially relevant studies independently by reading titles, abstracts and full texts. The studies were selected based on the following inclusion criteria: (1) the subjects were younger than or equal to 6 years old; (2) parental mental health including depression, anxiety and stress which was identified as the exposure factors in the literature; (3) the methods of the study were epidemiological studies (cross-sectional study, cohort study); (4) the criteria for diagnosing whether one child had caries was one or more primary tooth/teeth had present tissue loss and cavitation, missing or had been filled because of caries, which was in accordance with the standard proposed by the WHO or ICDAS; (5) the outcome indicators were odds ratio (OR), adjustment OR or Standardized mean differences (SMD) and 95% confidence interval (95% CI).

Exclusion criteria were: 1) duplicate publications; 2) inability to calculate ORs, SMD and 95% CI; 3) systematic reviews, meta-analyses and case reports.

The inquiry addressed in the present review was devised following the PICO model: “Whether children of parents with mental conditions more likely to develop ECC than those of mentally healthy parents?”.

- Participants: Children aged 0–6 years.
- Intervention: Parental mental health, including depression, anxiety, and stress (assessed using valid and reliable measures)
- Comparator: Parents with good mental health;
- Outcome: prevalence of ECC or dmft index

### Search strategy

Five electronic databases, PubMed, Web of Science, Embase, Ovid, and Cochrane Library, were searched up to October 2025. Hand search was performed in the reference lists of included studies. In all study phases, the articles were assessed by two researchers (LM and LJ) independently. A third researcher (HS) was consulted in cases of disagreement. The search strategies for each dataset were presented in Table 1.

**Table 1:** Database search indicators

Database	Search Strategies
PubMed	((“mental health”[MeSH Terms] OR (“health mental”[Title/Abstract] OR “mental hygiene”[Title/Abstract] OR “hygiene mental” [Title/Abstract])) AND “dental caries”[MeSH Terms]) OR (“caries dental”[Title/Abstract] OR “dental cavity”[Title/Abstract] OR “dental decay”[Title/Abstract] OR “carious lesions”[Title/Abstract] OR “carious dentin”[Title/Abstract] OR “dental white spot” [Title/Abstract])
Web of Science	Mental Health OR Health, Mental OR Mental Hygiene OR Hygiene, Mental (All Fields) and Dental Caries OR Caries, Dental OR Dental Cavity OR Dental Decay OR Carious Lesions OR Carious Dentin OR Dental White Spot (All Fields)
Embase	((‘mental health’:ab,ti OR ‘health, mental’:ab,ti OR ‘mental hygiene’:ab,ti OR ‘hygiene, mental’:ab,ti) AND (‘dental caries’:ab,ti OR ‘caries, dental’:ab,ti OR ‘dental cavity’:ab,ti OR ‘dental decay’:ab,ti OR ‘carious lesions’:ab,ti OR ‘carious dentin’:ab,ti OR ‘dental white spot’:ab,ti))
Ovid	((Mental Health OR Health, Mental OR Mental Hygiene OR Hygiene, Mental) and (Dental Caries OR Caries, Dental OR Dental Cavity OR Dental Decay OR Carious Lesions OR Carious Dentin OR Dental White Spot)).mp.[mp=ti, ab, tx, ct]
Cochrane Library	((‘mental health’:ab,ti OR ‘health, mental’:ab,ti OR ‘mental hygiene’:ab,ti OR ‘hygiene, mental’:ab,ti) and (‘dental caries’:ab,ti OR ‘caries, dental’:ab,ti OR ‘dental cavity’:ab,ti OR ‘dental decay’:ab,ti OR ‘carious lesions’:ab,ti OR ‘carious dentin’:ab,ti OR ‘dental white spot’:ab,ti))

### Study selection and data extraction

All the retrieved records were imported into EndNote X9 (Clarivate Analytics, Philadelphia, PA, USA) and duplicates were excluded. After removing duplicates, records were assessed based on title and abstract. Disagreement was solved by discussion, and when it was not possible, a third author (HS) was consulted. The collected data were authorship, publication year, study design, sample size, age, criteria for diagnosing caries in the primary dentition, assessment tools and scoring criteria for mental health, including depression, anxiety, and stress.

### Quality assessment

Two researchers (LM and LJ) independently assessed the methodological quality of the included studies, and any discrepancies were resolved by consultation with a third professor (HS) to reach a consensus. The Joanna Briggs Institute’s (JBI) evidence-based health care center’s checklist was used to evaluate the risk of bias in cross-sectional and cohort studies.The results of the assessment included “yes,” “no,” “unclear,” or “not applicable.”

In the quality assessment of analytical cross-sectional studies using the JBI scale (8 items), the risk of bias was categorized as follows: studies with six or more “Yes” responses are considered to have a low risk of bias; those with 4 to 5 “Yes” responses are regarded as having a moderate risk of bias; and only those with 0 to 3 “Yes” responses are classified as having a high risk of bias [28].

For cohort studies evaluated using the JBI scale (11 items), studies with eight or more “Yes” responses are considered to have a low risk of bias; those with 6 to 7 “Yes” responses are regarded as having a moderate risk of bias; and those with five or fewer “Yes” responses are classified as having a high risk of bias [29].

### Statistical analysis

Stata 16.0 (Stata Corporation, College Station, TX, USA) was used to perform the quantitative analysis. The SMD was calculated for numerical outcome and OR was calculated for dichotomous outcome. Heterogeneity across studies was assessed using the Cochrane Q Statistic (significance level at *P* < 0.10) and the I2 statistic [30, 31]. Heterogeneity was considered statistically insignificant if *P* > 0.10 and I2 ≤ 50%, and then the Mantel-Haenszel fixed-effect model (FEM) was used. Otherwise, the DerSimonian and Lairdrandom-effect model (REM) was used for combining the results [32]. Subgroups analyses were performed to explore possible sources of heterogeneity (according to type of parental mental disorder, study design, age range, dental caries diagnostic criteria and type of parent). The results of each meta-analysis were graphically represented in forest plots. The robustness of the analyses was assessed using sensitivity tests. Furthermore, Begg’s test and funnel plots were used to assess potential publication biases within the included studies [33]. All analysis used a 95% CI.

### Ethics approval

This study is a meta-analysis based exclusively on publicly available published literature. No original human or animal experimental data were collected or analyzed, and therefore, ethical approval was not required for this research.

## Results

### Search results

**Fig. 1:**
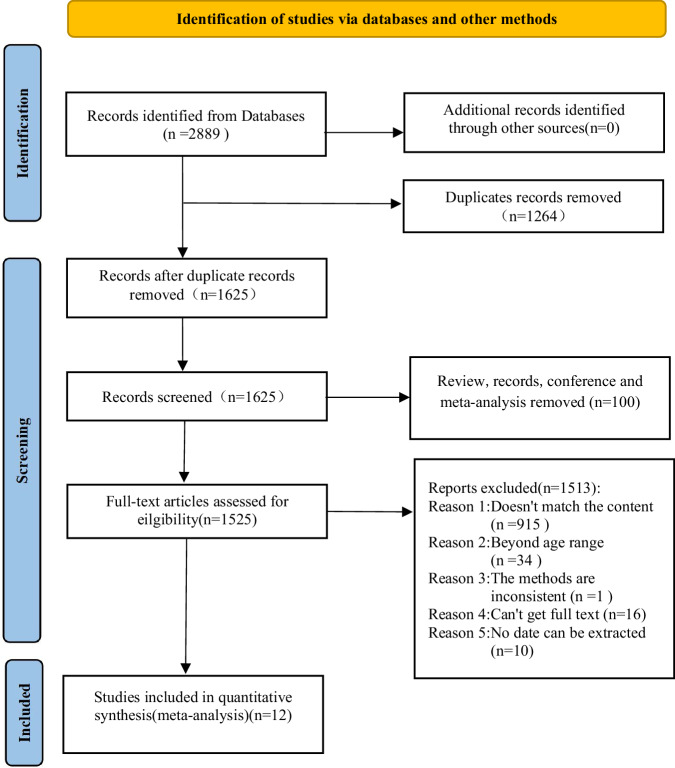
Search process based on the PRISMA flowchart. This figure illustrates the study selection process, including records identified from databases, removal of duplicates, screening, and final inclusion of 12 studies in the meta-analysis.

The PRISMA flowchart of study selection was shown in Fig. 1. Initial searches identified 2,889 records, from which 1,264 duplicates were removed. Title and abstract screening excluded 100 ineligible publications such as review reports. Full-text assessment excluded 1513 articles due to irrelevance, inability to extract data, or methodological inconsistencies, yielding 12 included studies [8–14, 16, 17, 19–21].

### Study characteristics

Characteristics of the selected studies are shown in Tables 2–4. Three out of the 12 articles studied the relationship between parental depression and ECC [8, 12, 17]. Seven articles explored the relationship between parental dental anxiety and ECC [8–11, 16, 20, 21]. Four studied the relationship between parental stress and ECC [8, 13, 14, 19]. Only one focused the relationship between parental general anxiety disorder and ECC [8]. Notably, the study by Alade et al. assessed all four outcome measures included in this meta-analysis[8]. Regarding of outcome indicators, the ECC prevalence rate was reported in 7 studies [8, 10, 12, 16, 17, 20, 34], while the dmft index was reported in 5 studies[9–11, 17, 21]. This meta-analysis included 12 studies, among which 9 focused on maternal mental health and 3 focused on parental mental health. Papers were published between 2001 and 2023. Of the included papers, nine studies were cross-sectional studies [8–11, 13, 14, 19–21], three studies were cohort studies [12, 16, 17].The total sample sizes of the included studies ranged from 110 [9] to 3645 [17] subjects. The age range of children in the included studies was 0-71 months. Eight studies used the WHO criteria [8, 10–12, 14, 19–21], while four studies adopted the ICDAS criteria for diagnosing ECC [9, 13, 16, 17].

**Table 2:** Main characteristics and the outcome regarding the ECC prevalence of the included studies.

Study ID	Age(N)	Study method	Case group (ECC/health)				Control group (ECC/health)				Caries diagnostic criteria	Mental health scale (cut-off)
			Anxiety	Depressive	DA	Stress	No mental illness					
AladeM,2021	6-71 m (1549)	cross sectional	11/300	15/359	6/143	14/351	55/1183	51/1124	60/1340	52/1132	WHO	GAD、MDAS、PSI、CESD (5、19、15、15)
Knoblauch,2019	3-4 y (120)	cross sectional									WHO	DAS (15)
Goettems,2012	2-5 y (608)	cross sectional									WHO	DAS (11)
Santos,2017	24-36 m (515)	cohort study		34/134				47/300			WHO	MINI Plus (NR)
Foxman (WestVirginia),2022	3 y (197)	cohort study			25/95				9/68		ICDAS	DAS (23)
Foxman (Pennsylvania),2022	3 y (382)	cohort study			15/246				7/112		ICDAS	DAS (23)
Fonseca,2023	0-48 m (3645)	cohort study		245/441				728/2231			ICDAS	EPDS (13)
Mahapatra,2022	3-5 y (342)	cross sectional				160/87				24/71	WHO	PSI (36)

*ECC* Early Childhood Caries, *DA* Dental anxiety, *ICDAS* International Caries Detection and Assessment System, *WHO* World Health Organization, *GAD* Generalized Anxiety Disorder scale, *MDAS* Modified Dental Anxiety Scale, *PSI* Parenting Stress Index, *CES-D* Centre for Epidemiologic Studies and Depression Scale, *DAS* Dental Anxiety Scale, *MINI Plus* Mini International Neuropsychiatric Interview, *EPDS* Edinburgh Postnatal Depression Scale, *NR* not reported.

**Table 3:** Main characteristics and the outcome regarding the dmft index of the included studies.

Study ID	Age(N)	Study method	Case group (mean ± sd)		Control group (mean ± sd)		Caries diagnostic criteria	Mental health scale (cut-off)
			DA	Depressive	No mental illness			
Gavic L (high DA),2022	6 y (110)	cross sectional	(4.00 ± 3.40)		(3.92 ± 3.47)		ICDAS	S-DAI (11)
Gavic L (mild DA),2022	6 y (210)	cross sectional	(4.00 ± 4.82)		(3.92 ± 3.47)		ICDAS	S-DAI (11)
Shruti,2018	3-6 y(227)	cross sectional	(6.16 ± 3.57)		(7.28 ± 3.85)		WHO	DAS (13)
Goettems (high DA),2012	2-5 y (498)	cross sectional	(1.88 ± 3.03)		(1.31 ± 2.51)		WHO	DAS (11)
Goettems (moderate DA),2012	2-5 y (472)	cross sectional	(1.97 ± 3.01)		(1.31 ± 2.51)		WHO	DAS (11)
Alhareky,2021	3-6 y (199)	cross sectional	(4.45 ± 3.92)		(2.56 ± 4.59)		WHO	MDAS (5)
Fonseca,2023	0-48 m(3645)	cross sectional		(2.50 ± 5.5)		(1.50 ± 4.4)	ICDAS	EPDS (13)

*dmft* decayed,missing and filled teeth, *ECC* Early Childhood Caries, *DA* Dental Anxiety, *ICDAS* International Caries Detection and Assessment System, *WHO* World Health Organization, *S-DAI* Short version of the Dental Anxiety Inventory, *DAS* Dental Anxiety Scale, *MDAS* Modified Dental Anxiety Scale, *EPDS* Edinburgh Postnatal Depression Scale.

**Table 4:** Main characteristics and the outcome regarding the adjusted ORs of the included studies.

Study ID	Age(N)	Study method	Corrected OR (95%CI)	Caries diagnostic criteria	Mental health scale (cut-off)
Finlayson (age 1–3 years),2007	1–3 y (446)	Cross sectional	0.62 (0.39–0.98)	ICDAS	PSI (NR)
Finlayson (age 4–5 years),2007	4–5 y (273)	Cross sectional	0.72 (0.50–0.03)	ICDAS	PSI (NR)
Quiñonez,2001	18–36 m (150)	C ross sectional	1.02 (0.99–1.05)	WHO	PSI (NR)

*OR* Odds Ratio, *95%CI* 95% Confidence Interval, *ECC* Early Childhood Caries, *ICDAS* International Caries Detection and Assessment System, *WHO* World Health Organization, *PSI* Parenting Stress Index, *NR* not reported.

The included studies assessed the parental mental health using screening instruments which were structured diagnostic interview according to ICD-10 criteria [35]. Four studies used the Dental Anxiety Scale (DAS) as the assessment instrument [10, 16, 20, 21], two studies employed the Modified Dental Anxiety Scale (MDAS) [8, 11] and one study utilized the Short version of the Dental Anxiety Inventory (S-DAI) [9] to evaluate parental dental anxiety. One study used the Center for Epidemiologic Studies Depression Scale (CES-D) [8], another used the Mini International Neuropsychiatric Interview Plus (MINI Plus) [12], and a third employed the Edinburgh Postnatal Depression Scale (EPDS) [17] to assess parental depression. Four studies used Parenting Stress Index (PSI) to assess the parent stress [8, 13, 14, 19]. One study used Generalized Anxiety Disorder scale (GAD) to identify the parent general anxiety disorder [8].

### Methodological quality appraisal of included studies

Table 5 shows the findings of the nine cross-sectional studies’ risk of bias assessments. Four studies received “yes (+)” for all 8 evaluation domains, indicating a low risk of bias [8–10, 20]. Five studies had limitations in 2 domains: the identification of confounding factors and description of confounding handling strategies [11, 13, 14, 19, 21]. Among these, three studies were rated “unclear (?)” [14, 19, 21]; two studies were rated “not applicable (×)”, but they were still classified as having a low risk of bias since they received 6 “Yes” responses [11, 13].

**Table 5:**
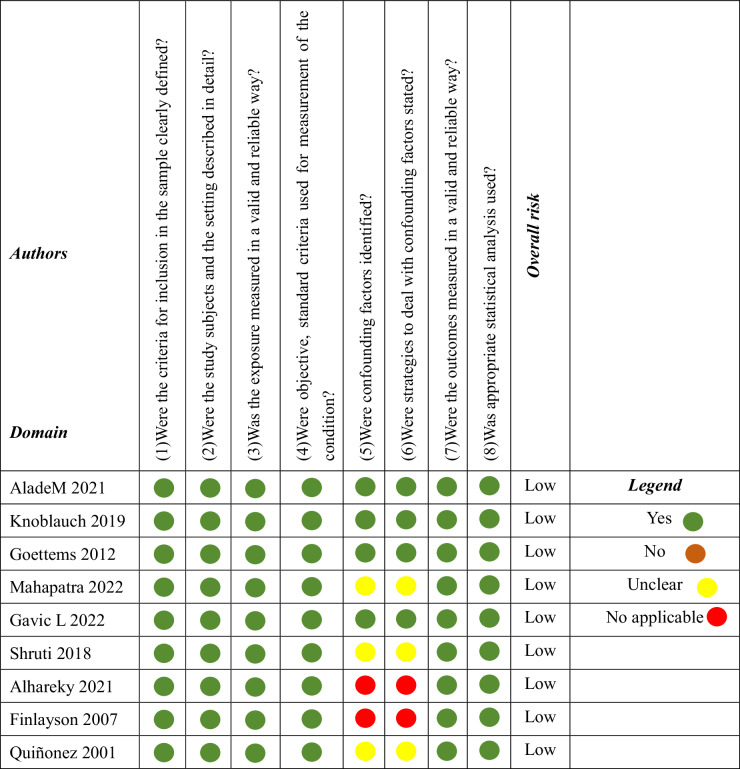
Summary of risk of bias evaluation for cross-sectional studies.

Table 6 shows the findings of the three cohort studies’ risk of bias assessments. One study failed to identify confounding factor, deal with confounding factors strategies and report followup time [12]; One study failed to identify confounding factor, deal with confounding factors strategies and address incomplete follow up [16]; One study failed to clarify baseline outcome status and address incomplete follow up [17]. Despite these limitations, the three studies which received 8 to 9 “Yes” responses, were classified as having a low risk of bias.

**Table 6:**
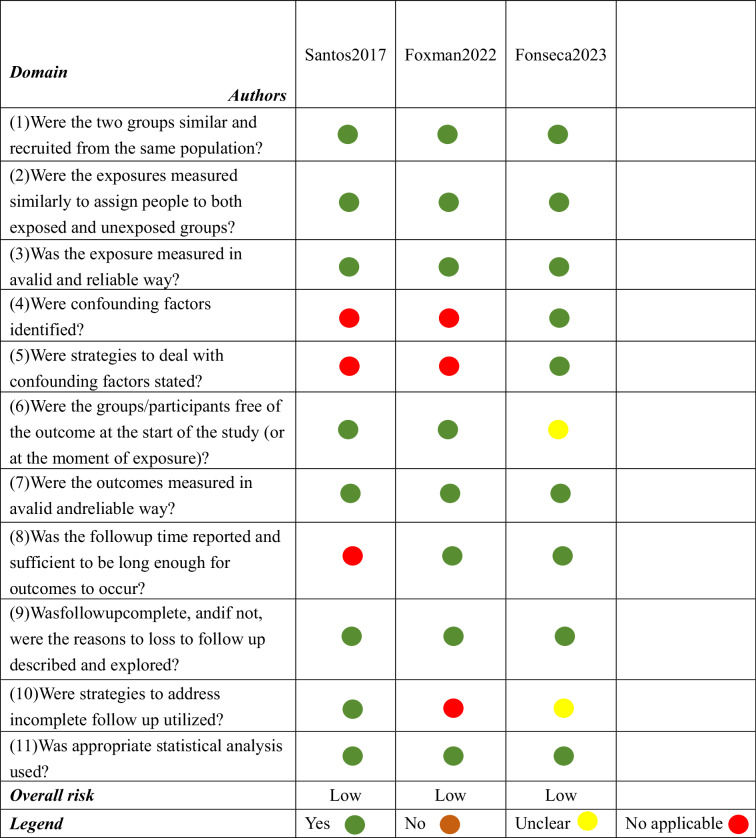
Summary of risk of bias evaluation for cohort studies.

### Meta-analysis results

**Fig. 2:**
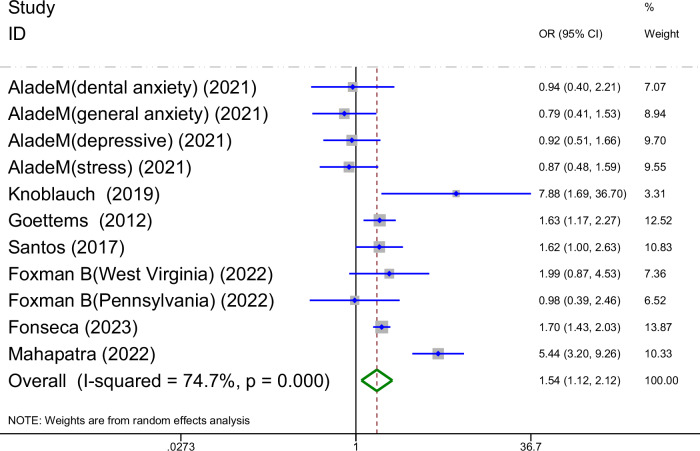
Forest plot of the impact of parental mental health on ECC prevalence (REM). This figure shows the pooled odds ratio (OR) and 95% confidence interval (CI) for the association between parental mental health and ECC prevalence, with significant heterogeneity observed (*I*^2^ = 74.7%, *9* ‹ 0.001).

A total of seven studies on the relationship between parental mental health and prevalence of ECC are shown in Fig. 2. The meta-analysis was conducted through the REM based on the result of heterogeneity(I^2^ = 74.7% > 50%, *P*_heterogeneity_ < 0.1). The meta-analysis results of the included studies showed that parental mental health was a risk factor for prevalence of ECC (OR = 1.54, 95%CI: 1.12-2.12, *P* = 0.008). Due to high heterogeneity, the subgroup analyses were conducted based on the type of parental mental conditions, study design, age range and dental caries diagnostic criteria.

**Fig. 3:**
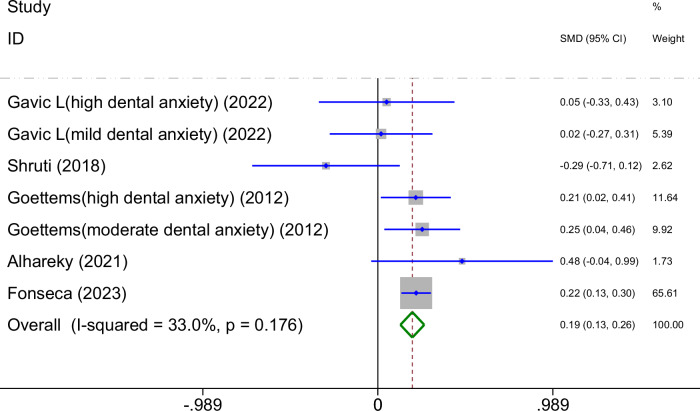
Forest plot of the impact of parental mental health on the dmft index in children (FEM). This figure presents the pooled standardized mean difference (SMD) and 95% confidence interval (CI) for the association between parental mental health and children’s dmft index, with low heterogeneity observed (*I*^2^ = 33.0%, *p* = 0.176).

A total of five studies on the relationship between parental mental health and dmft index are shown in Fig. 3. The meta-analysis was conducted through the FEM based on the low heterogeneity (I^2^ = 33.0% < 50%, *P*_heterogeneity_ > 0.1). The results indicated that poorer parental mental health was associated with a higher dmft index (SMD = 0.19, 95%CI: 0.13-0.26, *P* < 0.001).

### Subgroup analysis

#### Based on the type of parental mental conditions

**Fig. 4:**
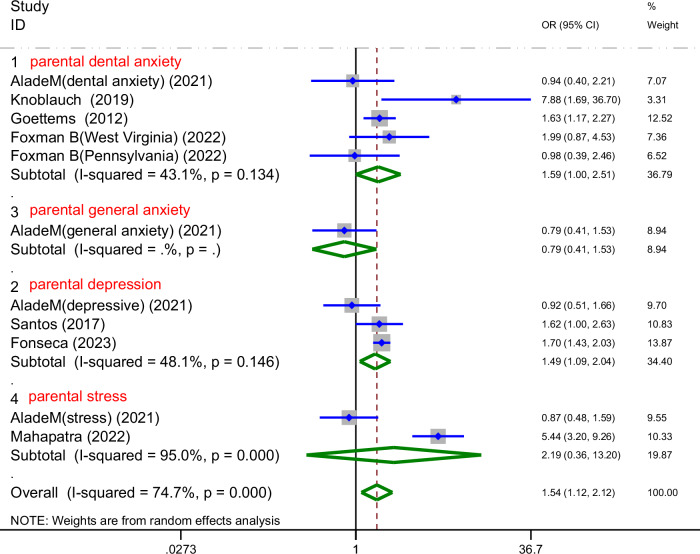
Forest plot of the subgroup analysis displaying the association between various parental psychiatric symptoms and ECC prevalence. This figure presents the subgroup analysis of the association between different parental mental health conditions (dental anxiety, general anxiety, depression, and stress) and ECC prevalence, showing pooled odds ratios (OR) and corresponding 95% confidence intervals (CI) for each subgroup.

**Fig. 5:**
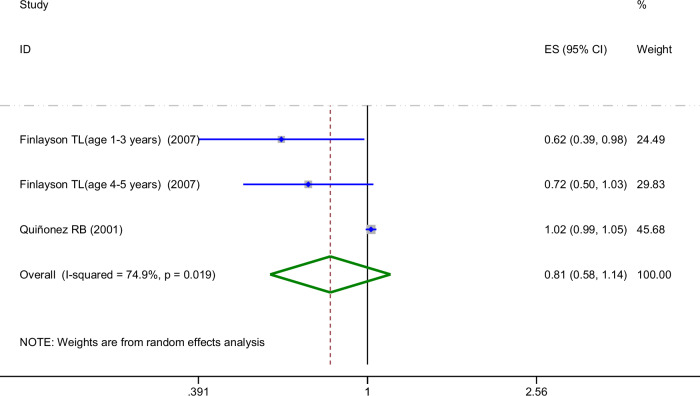
Forest plot of the association between parental stress (adjusted ORs) and ECC prevalence(REM). This figure presents the pooled effect size (ES) and 95% confidence interval (CI) for the association between parental stress (adjusted odds ratios) and ECC prevalence, with significant heterogeneity observed (*I*^2^ = 74.9%, *p* = 0.019).

As shown in Fig. 4, the four studies provided data regarding parental dental anxiety.The prevalence rate of ECC was 1.59 [95%CI: 1.00-2.51], *P* = 0.049. The meta-analysis revealed no significant heterogeneity (I^2^ = 43.1% < 50%, *P*_heterogeneity_ > 0.1). Three studies offered data concerning parental depression. The prevalence rate of ECC was 1.49 [95%CI: 1.09-2.04], *P* = 0.014. No significant heterogeneity was identified either (I^2^ = 48.1% < 50%, *P*_heterogeneity_ > 0.1). Two studies provided data regarding parental psychological stress.The prevalence rate of ECC was 2.19 [95%CI: 0.36-13.20], *P* = 0.394, I^2^ = 95.0% > 50%, *P*_heterogeneity_ < 0.1. Additionally, another two studies that provided adjusted ORs after controlling for confounding variables also focused on parental stress, with results as follows:0.81 [95%CI: 0.58-1.14], *P* = 0.227, I² = 74.9% > 50%, *P*_heterogeneity_ < 0.1 (Fig. 5). Since only one study describes parental general anxiety, no quantitative analysis were conducted. By performing a subgroup analysis, separating the studies based on the type of parental mental disorder, the heterogeneity was significantly reduced. Furthermore, the results indicated parental dental anxiety and depression were associated with the prevalence rate of ECC, while no association was observed for parental stress.

#### Based on the study design

These seven studies corresponded to the subgroup for ECC prevalence, 4 were cross-sectional studies. The heterogeneity was high (I² = 84.1%å 50%,*P*_heterogeneity_

**Fig. 6:**
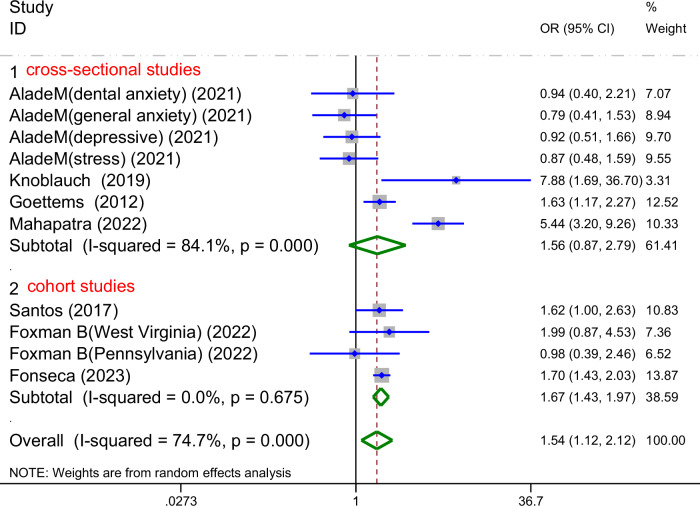
Forest plot of the subgroup analysis based on the study design. This figure presents the subgroup analysis of the association between parental mental health and ECC prevalence according to study design (cross-sectional vs. cohort studies), showing pooled odds ratios (OR) and 95% confidence intervals (CI) for each subgroup, with significant heterogeneity observed in the cross-sectional subgroup (*I*^2^ = 84.1%, *p* = 0.000) and low heterogeneity in the cohort subgroup (*I*^2^ = 0.0%, *p* = 0.675).

<0.1). Meanwhile, among the 3 cohort studies, no heterogeneity was detected (I² = 0% < 50%, *P*_heterogeneity_å 0.1)(Fig. 6).

#### Based on the age range

**Fig. 7:**
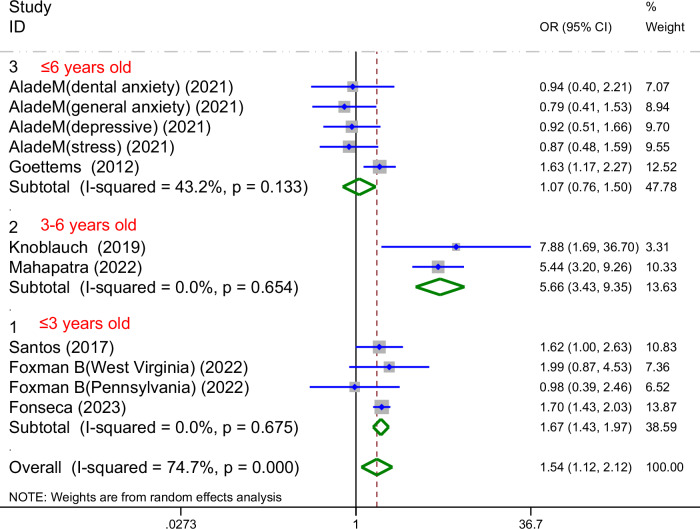
Forest plot of the subgroup analysis based on the age range. This figure presents the subgroup analysis of the association between parental mental health and ECC prevalence according to children’s age range (≤3 years, 3–6 years, and ≤6 years old), showing pooled odds ratios (OR) and 95% confidence intervals (CI) for each subgroup, with low to moderate heterogeneity observed within subgroups (*I*^2^ ranging from 0.0% to 43.2%).

All analyses within this subgroup were based on the seven studies for ECC prevalence, and 3 focused on participants aged 3 years or younger, 2 on those aged 3-6 years, and 2 on those aged 6 years or younger. No heterogeneity was found in the subgroup analysis of children aged 3 years or younger and those aged 3-6 years(I² = 0%). A low heterogeneity was observed in the subgroup analysis of children aged 6 years or younger (I² = 43.2%) (Fig. 7). Heterogeneity was significantly reduced following subgroup analysis.

#### Based on the dental caries diagnostic criteria

**Fig. 8:**
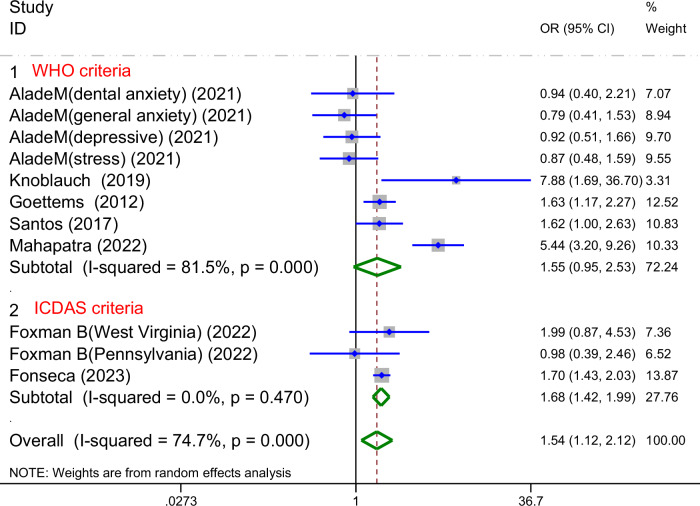
Forest plot of the subgroup analysis based on the dental caries diagnostic criteria. This figure presents the subgroup analysis of the association between parental mental health and ECC prevalence according to dental caries diagnostic criteria (WHO vs ICDAS), showing pooled odds ratios (OR) and 95% confidence intervals (CI) for each subgroup. High heterogeneity was observed in the WHO criteria subgroup (*I*^2^ = 81.5%, *p*= 0.000), while no significant heterogeneity was present in the ICDAS criteria subgroup (*I*^2^ = 0.0%, *p* = 0.470).

Using the same seven studies from the ECC prevalence subgroup, we performed a further analysis stratified by diagnostic criteria (WHO criteria vs. ICDAS criteria). Significant heterogeneity remained across the 5 studies using the WHO criteria (I² = 81.5%). No heterogeneity was observed among the 2 studies adopting ICDAS criteria (I² = 0%) (Fig. 8).

#### Based on the parental type

**Fig. 9:**
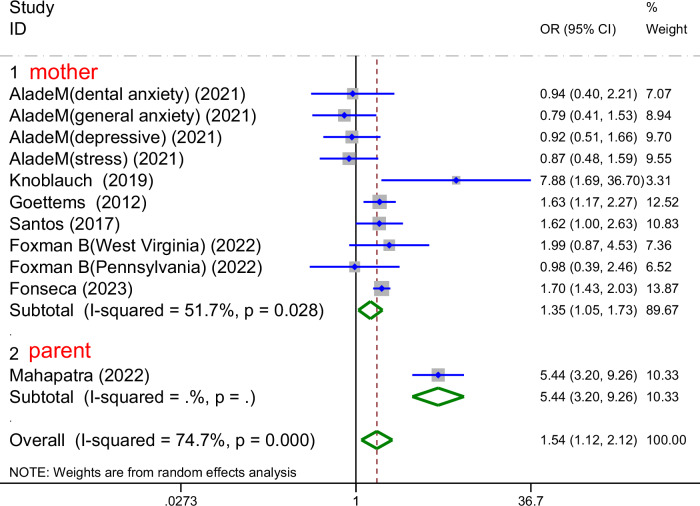
Forest plot of the subgroup analysis bas on  the parental type. This figure presents the subgroup analysis of the association between parental mental health and ECC prevalence according to parental type (mother vs parent), showing pooled odds ratios (OR) and 95% confidence intervals (CI) for each subgroup. Moderate heterogeneity was observed in the mother subgroup (*I*^2^ = 51.7%, *p* = 0.028), while the parent subgroup showed no significant heterogeneity (*I*^2^ not applicable, single study).

Among the 7 studies included in the subgroup analysis for ECC prevalence, a subgroup analysis stratified by parental type was performed (Fig. 9). In 6 studies focusing on maternal mental health, poor maternal mental health was significantly associated with an increased risk of ECC (I^2^ = 51.7%, *P*_heterogeneity_ < 0.1, OR = 1.35, 95% CI: 1.05-1.73, *P* = 0.028). One study included both parents, however, quantitative pooling was not conducted due to the limited number of studies. Following subgroup stratification, between-study heterogeneity was notably decreased, suggesting that parental type may be an important source of heterogeneity and can help reveal the different effects of parental mental health on early childhood caries.

### Sensitivity analysis

Sensitivity analysis was conducted by omitting one study at each time and recalculating the pooled results. Among the studies about parental mental health and ECC prevalence, the overall risk estimation did not vary materially with a range from 1.51 (95%CI: 1.08-2.11) to 1.90 (95%CI: 1.28-2.83). The sensitivity analysis results of the relationship between parental mental health and dmft index showed that the risk estimation range slightly changed from 0.19 (95%CI: 0.12-0.26) to 0.21 (95%CI: 0.14-0.28), indicating that the results were stable.

### Publication bias

**Fig. 10:**
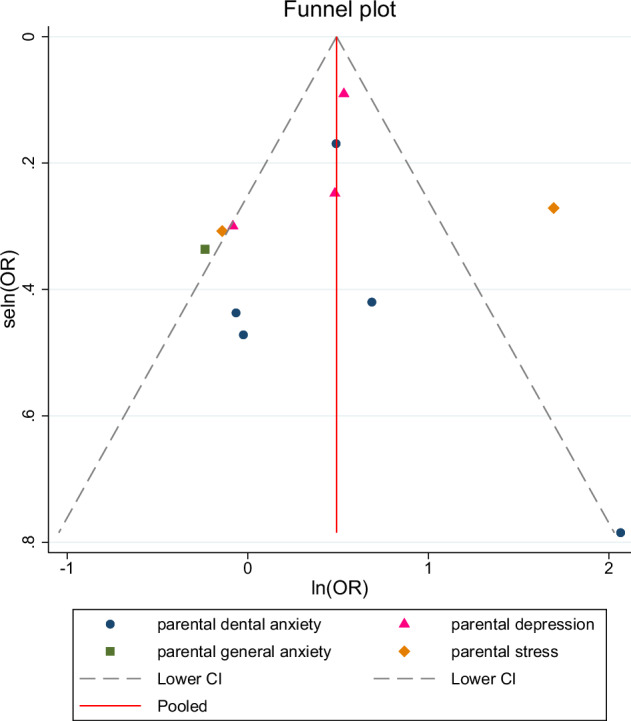
Funnel plot displaying the association between parental mental health and ECC prevalence. This figure presents the funnel plot assessing publication bias for the association between parental mental health and ECC prevalence. The plot displays the natural logarithm of the odds ratio (lnOR) against its standard error (selnOR), with the pooled effect estimate shown by the red line. Different symbols represent subgroups of parental mental health conditions (dental anxiety, general anxiety, depression, and stress).

**Fig. 11:**
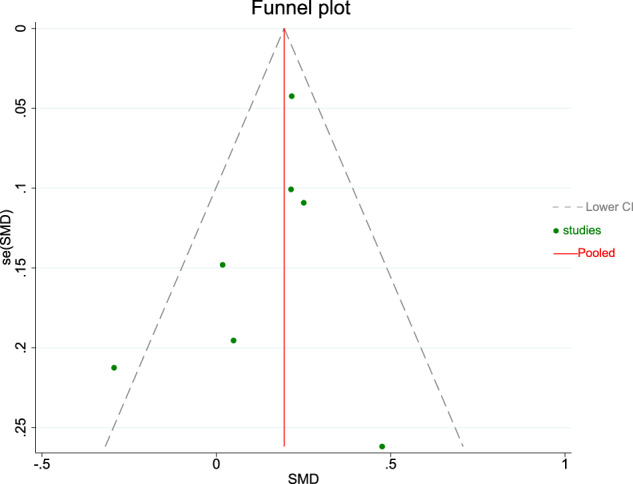
Funnel plot displaying the association between parental mental health and dmft index. This figure presents the funnel plot assessing publication bias for the association between parental mental health and the dmft index. The plot displays the standardised mean difference (SMD) against its standard error (se(SMD)), with the pooled effect estimate shown by the red line. Green dots represent individual included studies.

An assessment of the publication bias for included studies was made by the Begg’s test (parental mental health and ECC prevalence: *P* = 0.12 > 0.1; parental mental health and dmft index: *P* = 0.55 > 0.1) respectively, and the funnel plots are illustrated in Figs. 10 and 11 which no asymmetry emerges.

## Discussion

This meta-analysis included 12 original studies published before October 2025 that investigated the relationship between parental mental health and ECC, with a total of 10,329 children aged 0-6 years enrolled. Quality assessment of the included studies showed low risk of bias. The results of the meta-analysis provide evidence that children of parents with mental conditions had a significantly higher prevalence rate of ECC and higher dmft index than those without mental conditions. Furthermore, subgroup analysis revealed a significant association between parental dental anxiety, depression and ECC, whereas stress was not observed. Subgroup analyses performed to explore the sources of heterogeneity revealed that the type of parental mental conditions, study design, age range, and dental caries diagnostic criteria were the sources of heterogeneity. A 2020 systematic review by Marcia of 7 studies linked maternal depression and anxiety to caries in children aged 0-12 [25]. However, this meta-analysis has a broader scope, encompassing mental health in both fathers and mothers and examining their association with ECC prevalence and dmft index. To date, this is the most comprehensive meta-analysis and systematic review that explored this relationship. This meta-analysis provides a rational basis for the further prevention and control of ECC (Fig. 11).

This meta-analysis indicated that parental mental conditions constitute a risk factor for both the prevalence and severity of ECC. Building on this, recent studies by Hashimoto, Fang, Ghanim, Andreola, and colleagues have further analyzed the potential etiological mechanisms linking parental mental conditions to ECC from microbiological and physiological developmental perspectives [36–39]. From a microbiological perspective, Hashimoto has confirmed that mental conditions can disturb the oral microbial homeostasis, thereby increasing the risk of caries and periodontitis in affected individuals [36]. Fang et al. believed that in the context of chronic parental mental conditions, the abundance of cariogenic bacteria in the parental oral cavity may be altered due to the disturbed microbial balance; these bacteria can then be transmitted to children through salivary contact, such as sharing food or utensils. Given the immaturity of children’s immune systems and underdeveloped oral defense mechanisms, they are more susceptible to the colonization of these cariogenic bacteria, which may further increase their risk of developing ECC [37], consistent with the findings of the present study. From a physiological development standpoint, Ghanim et al. pointed out that enamel formation of deciduous and permanent teeth starts at the 14th and 28th gestational weeks, respectively; maternal stress during this critical period may disrupt ameloblast function, which is associated with enamel developmental defects like hypoplasia and hypomineralization and increased caries susceptibility [38]. What’s more, additionally, Andreola has indicated that children of parents with mental conditions may face a higher risk of malnutrition, which can promote the development of ECC through dual pathways [39]. Specifically, deficiencies in nutrients such as vitamin D, iron, calcium, and albumin may be associated with defective enamel formation, resulting in an acid-susceptible and plaque-retentive tooth surface [40]. Concurrently, malnutrition can impair the exocrine system, and salivary gland dysfunction may compromise oral immunity and the ability to neutralize plaque acids, thereby further increasing caries risk [41]. Collectively, these findings suggest that vigilant monitoring and proactive management of parental mental health may be a key strategy for reducing ECC risk.

This subgroup analysis further confirmed a strong association between ECC prevalence and parental mental conditions, namely dental anxiety and depression. A growing body of evidence indicated that parental dental anxiety and depression elevate children’s caries risk, mediated by detrimental caregiving practices [37, 42, 43]. Key behavioral pathways include a compromised adherence to essential oral hygiene protocols, such as daily tooth brushing, fluoride use, routine check-ups and dietary regulation [39, 44]. These factors collectively facilitate plaque accumulation and increase the susceptibility to caries in dependent children directly [45]. Furthermore, parental avoidance of dental visits and negative attitudes toward oral health can be modeled by children, diminishing the child’s own motivation and proactivity in performing self-care [46]. The aforementioned reasons related to caregiving, diet, and learned behaviors solidify the established relationship between parental mental conditions and the prevalence of ECC.

Additionally, our subgroup analysis founded no significant association between parental stress and ECC, a finding corroborated by the adjusted odds ratios (ORs) obtained after controlling for potential confounders [13, 14]. To date, the relationship between parental stress and ECC remains inconclusive. Contrary to findings showing no association [47], some studies suggested a complex link. Stress, particularly among highly-educated or high-income parents, may correlate with greater health awareness and responsibility, leading to preventive oral care practices that lower ECC risk [13]. However, an opposing perspective, as held by Menon, contended that high parental stress directly compromises oral health supervision, significantly increasing ECC risk [16]. We noted a diversity of stress scales in the existing literature. To facilitate comparison and synthesis, future studies should use a standardized tool and focus on elucidating the mechanisms, specifically exploring psychosocial pathways involving socioeconomic status, parental education, and family income.

The Q-test indicated a high degree of heterogeneity in this meta-analysis. In the subgroup analysis, heterogeneity was reduced. We identified three reasons contributing to the heterogeneity. Firstly, the study lacked strict temporal delineation, as it included parents with mental conditions during pregnancy, the perinatal period, and the postpartum period-a span during which mental health status is dynamic. Secondly, geographical and economic disparities introduced variability, as regions with poorer economic development typically exhibit higher ECC prevalence. Thirdly, the methods for clinical caries examination were inconsistent, ranging from visual inspection alone to visual inspection with probing and cleaning; the less rigorous methods may have compromised diagnostic accuracy. Nevertheless, the findings of this study are reliable deriving from high-quality literature. The robustness was confirmed by a stable sensitivity analysis and Begg’s test showed no significant publication bias, further corroborating the strength of the evidence.

This study has several potential limitations. To begin with, the assessment of parental mental health, based on a variety of self-reported questionnaires, is subject to recall bias, which may potentially compromise the accuracy of the results. Additionally, inclusion of primarily cross-sectional studies prevents causal inference. Prospective cohort studies are recommended for future investigations.

## Conclusion

This meta-analysis confirmed a relationship parental mental health and ECC, specifically parental dental anxiety and depression. The more severe the parental dental anxiety and depression, the higher the prevalence of ECC and the dmft index. However, the relationship between psychological stress and ECC was not observed.

## Data Availability

The data supporting the findings of this meta-analysis are available within the article. All data were extracted from previously published, publicly available original studies, which have been appropriately cited in the reference list.
